# Eicosanoid and cytokine levels in plasma of patients during mesenteric infarction

**DOI:** 10.1080/09629359791983

**Published:** 1997-02

**Authors:** N. Nathan, Y. Denizot, P. Feiss

**Affiliations:** 1 Department of Anaesthesia CHU Dupuytren 2 Avenue Martin Luther King Limoges 87042 France; 2 Laboratoire d'Hématologie Expérimentale Faculté de Médecine 2 Rue du Dr Marcland Limoges Cédex 87025 France

## Abstract

Multible organ failure (MOF) induced by mesenteric infarction is associated with a high mortality rate. This study reports eicosanoid and cytokine levels in the blood of three atherosclerotic patients who ultimately died from MOF induced by mesenteric infarction. High plasma levels of 6- keto-prostaglandin (PG) F_1α_ (the stable metabolite of PGI_2_), interleukin (IL)-6 and IL-8 are observed whereas plasma tumour necrosis factor alpha (TNFα), TxB_2_ (the stable metabolite of TxA_2_), PGE_2_, leukotrienes (LT)B_4_ and LTC_4_, and whole blood platelet-activating factor levels are not different from values obtained in similarly severe atherosclerotic patients. This short report questioned the clinical involvement of TNFα during such a pathology where a persistent translocation of endotoxin has been observed through the gut endothelial barrier. Activation of phospholipase A_2_ is suggested by the increase in the stable metabolite of PGI_2_ and might be by itself or through lipidic metabolites, a major systemic stimulus of IL-6 and IL-8 production.


Mediators of Inflammation, 6, 75–77 (1997)

### Eicosanoid and cytokine levels in plasma of patients during mesenteric infarction

N. Nathan,[1,CA] Y. Denizot[2] and P. Feiss[1]

[1]Department of Anaesthesia, CHU Dupuytren, 2 Avenue Martin Luther King, 87042 Limoges, France; and [2]Laboratoire d'Hématologie Expérimentale, Faculté de Médecine, 2 Rue du Dr Marcland, 87025 Limoges Cédex, France

[CA]Corresponding Author
Fax: (+33) 05 55 05 67 92

**MULTIPLE organ failure (MOF) induced by mesenteric infarction is associated with a high mortality rate. This study reports eicosanoid and cytokine levels in the blood of three atherosclerotic patients who ultimately died from MOF induced by mesenteric infarction. High plasma levels of 6-keto-prostaglandin (PG) $F_{1\alpha}$ (the stable metabolite of $PGI_2$), interleukin (IL)-6 and IL-8 are observed whereas plasma tumour necrosis factor alpha (TNFα), $TxB_2$ (the stable metabolite of $TxA_2$), $PGE_2$, leukotrienes (LT)$B_4$ and $LTC_4$, and whole blood platelet-activating factor levels are not different from values obtained in similarly severe atherosclerotic patients. This short report questioned the clinical involvement of TNFα during such a pathology where a persistent translocation of endotoxin has been observed through the gut endothelial barrier. Activation of phospholipase $A_2$ is suggested by the increase in the stable metabolite of $PGI_2$ and might be by itself or through lipidic metabolites, a major systemic stimulus of IL-6 and IL-8 production.**

**Key words:** Interleukin, Leukotriene, Mesenteric infarction, Prostaglandin, Tumour necrosis factor alpha

### Introduction

Mesenteric infarction is associated with a high mortality rate. Lactic acidosis, hyperthermia, acute respiratory distress syndrome and cardiogenic shock are common consequences of this disease suggesting the plasma release of factors inducing multiple organ failure (MOF). Eicosanoids [such as prostaglandins (PG) $E_2$ and $I_2$, leukotrienes (LT) $B_4$ and $C_4$ and platelet-activating factor (PAF)] and cytokines [such as tumour necrosis factor alpha (TNFα), interleukin (IL)-6 and IL-8] could be involved in this process because of their cardiovascular and proinflammatory effects. Phospholipase $A_2$ (the enzyme leading to eicosanoid production) is stimulated in experimental mesenteric ischaemia-reperfusion syndrome.[1] On the other hand, in animal and human mesenteric ischaemia, the bacterial translocation might trigger TNFα then IL-6 and IL-8 plasma release.[2] Finally, the production and action of cytokines and eicosanoids are linked to each other. Thus we have assessed eicosanoid and cytokine plasma levels in three patients with mesenteric infarction before MOF development compared with 10 atherosclerotic patients.

### Patients and Methods

The first patient (71 years old) developed a jejunal infarction 48 h after a transurethral resection of the prostate which was complicated by myocardial ischaemia and supraventricular arrhythmia. Treatment included anti-arrhythmic drugs (amiodarone and deslanoside), and venous thrombosis prophylaxis with a low molecular weight heparin (dalteparine 5000 IU). At blood sampling, blood pressure (120/80 mmHg) and heart rate (100 cpm) were stable but hyperthermia (38°C), hyperleukocytosis (13 g.l$^{-1}$) and metabolic acidosis (pH = 7.31, $HCO_3^-$ = 18.1 mmol.l$^{-1}$) were present. The prothrombin time (10 s) and the platelet count (359 g.l$^{-1}$) were normal. A cardiogenic shock occurred in the early postoperative period and the patient died 34 days later of a MOF syndrome.

The second patient (87 years old) suffered from angina pectoris, non-insulin-dependent diabetes mellitus and chronic obstructive pulmonary disease. His right leg had been amputed 2 days before and he was taking orally a calcium inhibitor (nifedipine) and transdermal trinitrine. At blood sampling arterial pressure

© 1997 Rapid Science Publishers

(120/80 mmHg) and heart rate (85 cpm) were stable but hyperthermia (38.5°C), polypnoea, and severe lactic acidosis (pH = 7.15, $HCO_3^-$ = 8.8 mmol.l$^{-1}$, blood lactates = 14.4 mmol.l$^{-1}$) were present. Activated partial thrombin time (30.7 s), prothrombin time (14 s) and platelet count (257 g.l$^{-1}$) were normal. Surgery revealed a partial small bowel necrosis without occlusion of the mesenteric artery and vein. The patient died 19 h later from a severe cardiogenic shock.

The third patient (76 years old) had a mitral prosthesis and atria fibrillation treated by oral anticoagulant (fluindone). At blood sampling, blood pressure (150/80 mmHg) and heart rate (105 cpm) were stable but associated with hyperthermia (39°C), metabolic acidosis (pH = 7.38, $HCO_3^-$ = 16 mmol.l$^{-1}$) and hyperleukocytosis (25.5 g.l$^{-1}$). Coagulation parameters demonstrated the biological efficiency of the anticoagulant treatment without associated disseminated intravascular coagulation (prothrombin time$_{INR}$ = 19%, factor V level = 133%). Laparotomy revealed a necrosis of the whole digestive tract and the patient died 19 h later from cardiogenic shock.

The control patients (mean age 69 [26] years) (eight males/two females) suffered from a severe atherosclerotic disease and were scheduled for coronary artery bypass grafting. Their treatment included trinitrine or calcium inhibitors. All patients had their aspirin and angiotensin converting enzyme inhibitors stopped for 1 week and 3 days respectively at the time of blood sampling for which they gave written informed consent.

Blood samples were obtained at the time of diagnosis of mesenteric infarction during laparotomy before MOF occurred. Blood PAF was ethanol extracted, processed and assayed by platelet aggregation.[3] Plasma PAF acetylhydrolase activity (the enzyme which inactivates PAF) was measured by the degradation of $^3$H-PAF.[4] Cytokines and other eicosanoids were determined in plasma with specific enzymo-immunosorbent assay (EIA) after a three- and five-fold dilution in EIA buffer (TEBU and Cayman Chemicals, sensitivity 2 and 5 pg.ml$^{-1}$, respectively). The results (shown as individual values and median [range] for the control group) are compared with those of the control patient by a Mann–Whitney *U*-test.

### Results and Discussion

The results are presented in Table 1. High plasma IL-6, IL-8 and 6-keto-PGF$_{1\alpha}$ (the stable metabolite of $PGI_2$) levels were found in these three mesenteric infarcted patients suggesting a role for these mediators in the MOF syndrome which occurred later. High IL-6 and IL-8 levels are associated with a poor prognosis during sepsis syndrome where TNF$\alpha$ is supposed to trigger their synthesis. In these three patients, TNF$\alpha$ was not detectable suggesting that its production was not clinically relevant because it was not as high as during sepsis despite a theoretical persistent endotoxic stimulus.[2] TNF$\alpha$ independent pathway of IL-6 and IL-8 synthesis have already been described during sepsis as well as during experimentally induced cancer. However, the role of TNF$\alpha$ as a mediator inducing IL-6 and IL-8 synthesis cannot be definitively excluded. Indeed, the fixation of TNF$\alpha$ on soluble receptors and/or its transient release may lead to difficulties in its detection in blood. No elevated blood PAF levels were observed in these patients while PAF is produced by the intestinal tract after experimental hypoxia and PAF antagonists reduced mesenteric ischaemia-induced mortality in animals.[5] These low PAF levels did not result from an increased PAF degradation as shown by the

**Table 1.** Blood platelet-activating factor (PAF), plasma TNF$\alpha$, IL-6, IL-8, $LTB_4$, $LTC_4$, 6-keto-PGF$_{1\alpha}$, $TxB_2$, $PGE_2$ (pg.ml$^{-1}$) and acetylhydrolase activity (nmol.min$^{-1}$.ml$^{-1}$) in patients with mesenteric infarction (individual values) compared with atherosclerotic patients (median [range]) by Mann–Whitney U-tests

| | Patient 1 | Patient 2 | Patient 3 | Control patients (n = 10) | *P* |
|---|---|---|---|---|---|
| TNF$\alpha$ | 0 | 0 | 0 | 0 [0] | 1 |
| IL-6 | 980 | 3800 | 600 | 0 [18] | 0.01 |
| IL-8 | 160 | 210 | 225 | 36 [135] | 0.01 |
| PAF | 175 | 220 | 50 | 113 [49] | 0.55 |
| Acetylhydrolase | 10.4 | 51.7 | 36.0 | 65.9 [49.3] | 0.35 |
| 6 keto PGF$_{1\alpha}$ | 3400 | 2600 | 2300 | 240 [620] | 0.01 |
| $TxB_2$ | 125 | 450 | 5 | 72.5 [500] | 0.67 |
| $PGE_2$ | 760 | 180 | 1880 | 220 [848] | 0.35 |
| $LTB_4$ | 0 | 0 | 0 | 0 [0] | 0.32 |
| $LTC_4$ | 0 | 0 | 0 | 0 [128] | 0.26 |

normal PAF acetylhydrolase activity. The other lipidic mediators were not detectable or were at normal levels except for the stable metabolite of $PGI_2$ which may be secreted chiefly from vascular endothelium after hypoxia. The levels of $TxB_2$ in plasma were similar to those of severe atherosclerotic patients. $TxA_2$ plasma levels are supposed to be originated chiefly from activated platelets. The absence of haemostasis impairment in our patients may, in part, explain the similar values of their $TxB_2$ blood levels compared with those of atherosclerotic patients. $PGI_2$ is a potent vasodilator whereas $TxA_2$ highly vasoconstricts peripheral vessels. The high $PGI_2$–$TxB_2$ ratio observed in our patient could be considered as a protective mechanism to counteract the mesenteric hypoperfusion.

The number of subjects in this study is small due to difficulties in obtaining blood before MOF occurrence. Indeed, the diagnosis of mesenteric infarction is usually made late, at the time of MOF. Nevertheless our results confirm experimental data questioning the true role of TNF$\alpha$ induced by endotoxinaemia as a trigger for inflammatory reaction and MOF as it has already been observed during sepsis.[6] The activation of phospholipase $A_2$ and the subsequent release of eicosanoids which may in turn stimulate IL-6 or IL-8 production are other ways of research deserving further investigation in humans.

### References

1. Koike K, Moore EE, Kim FJW, Carl WS, Banerjee A. Gut phospholipase $A_2$ mediates neutrophil priming and lung injury after mesenteric ischaemia-reperfusion. *Am J Physiol* 1995; **268**: G397–G403.
2. Gogler H, Meckes P, Beger HG. Endotoxin bei diffus-eitriger peritonitis. *Zentralbl Chir* 1985; **110**: 1388–1398.
3. Denizot Y, Trimoreau F, Dupuis F, Verger C, Praloran V. PAF and hematopoiesis III. Presence and metabolism of platelet-activating factor in human bone marrow. *Biochem Biophys Acta* 1995; **1265**: 55–60.
4. Miwa M, Miyaka T, Yamanaka T, Sugatani J, Suzuki Y, Sakata S, Araki Y, Matsumoto M. Characterization of serum platelet-activating factor (paf) acetylhydrolase. *J Clin Invest* 1988; **82**: 1983–1991.
5. Filep JG, Braquet P, Mozes T. Interactions between platelet-activating factor and prostanoids during ischemia-reperfusion-induced shock in the anesthetized dog. *Circ Shock* 1991; **35**: 1–8.
6. Koike K, Moore EE, Moore FA, Read RA, Carl VS, Banerjee A. Gut ischemia/reperfusion produces lung injury independent of endotoxin. *Crit Care Med* 1994; **22**: 1438–1444.

**Received 13 November 1996; accepted 5 December 1996**
